# A RNA-DNA Hybrid Aptamer for Nanoparticle-Based Prostate Tumor Targeted Drug Delivery

**DOI:** 10.3390/ijms17030380

**Published:** 2016-03-14

**Authors:** John C. Leach, Andrew Wang, Kaiming Ye, Sha Jin

**Affiliations:** 1 Department of Biomedical Engineering, College of Engineering, University of Arkansas, Fayetteville, AR 72701, USA; jcleach@uams.edu (J.C.L.); kye@binghamton.edu (K.Y.); 2 Ocean Nanotech, 2143 Worth Lane, Springdale, AR 72764, USA; awang@oceannanotech.com; 3 Department of Biomedical Engineering, Thomas J. Watson School of Engineering and Applied Sciences, State University of New York in Binghamton, Binghamton, NY 13902, USA

**Keywords:** aptamer, nanoparticle, prostate cancer, targeted drug delivery

## Abstract

The side effects of radio- and chemo-therapy pose long-term challenges on a cancer patient’s health. It is, therefore, highly desirable to develop more effective therapies that can specifically target carcinoma cells without damaging normal and healthy cells. Tremendous efforts have been made in the past to develop targeted drug delivery systems for solid cancer treatment. In this study, a new aptamer, A10-3-J1, which recognizes the extracellular domain of the prostate specific membrane antigen (PSMA), was designed. A super paramagnetic iron oxide nanoparticle-aptamer-doxorubicin (SPIO-Apt-Dox) was fabricated and employed as a targeted drug delivery platform for cancer therapy. This DNA RNA hybridized aptamer antitumor agent was able to enhance the cytotoxicity of targeted cells while minimizing collateral damage to non-targeted cells. This SPIO-Apt-Dox nanoparticle has specificity to PSMA^+^ prostate cancer cells. Aptamer inhibited nonspecific uptake of membrane-permeable doxorubic to the non-target cells, leading to reduced untargeted cytotoxicity and endocytic uptake while enhancing targeted cytotoxicity and endocytic uptake. The experimental results indicate that the drug delivery platform can yield statistically significant effectiveness being more cytotoxic to the targeted cells as opposed to the non-targeted cells.

## 1. Introduction

Cancer is still the number two leading cause of death, despite advances in surgery, radiotherapy, and chemotherapy treatment [1,2]. Chemotherapy and radiotherapy are powerful, but these treatments’ side effects not only pose long-term challenges on the patients’ quality of life, but also increase mortality and limit the opportunity of further treatment [3]. For instance, a commonly used anticancer drug, doxorubicin, can cause congestive heart failure and dilated cardiomyopathy when it is nonspecifically absorbed by non-targeted tissues, such as those of the cardiovascular system [4]. The severity of complications from these antineoplastics is directly proportional to the dosage and administration methods [4]. To eliminate these side effects, these drugs must be delivered specifically to targeted tumors at a minimum dose.

Numerous efforts have been made so far to develop targeted drug delivery systems for cancer treatment [5,6]. The general idea for targeted drug delivery is to combine anti-cancer drugs with a molecular recognition element that can specifically interact with tumor-associated antigens or receptors. Interaction between the added molecular recognition element and the tumor cells ensures the delivery of anti-cancer drugs elusively to tumors. The use of antibodies for targeting specific cancer antigens has been a major focus of studies in the past decades, and only recently has there been much innovative progress. With the advent of the systematic evolution of ligands through exponential enrichment procedures, a new class of targeting-capable biomolecules, *i.e.*, aptamers, emerged [7,8].

Aptamers, relatively short single-stranded DNA or RNA oligonucleotides capable of mimicking the antigen specificity of an antibody [9,10], have shown to possess a very high specificity for drug delivery in cancer chemotherapy [11,12]. They can provide reduced expense, minimize immunogenic response, and enable easy *in vitro* synthesis [7,10]. Unlike antibodies, aptamers can be chemically synthesized and produced at a low cost. Moreover, they can be readily designed or chemically evolved to either increase or relax their affinity for a particular target molecule, such as a cancer antigen. For instance, SELEX (systematic evolution of ligands by exponential enrichment) has been developed and used for evolving and selecting a particular DNA or RNA aptamer that has a desired affinity for a cancer antigen of interest [13]. A number of therapeutic and sensing aptamers have been discovered this way [14,15]. These advantages make them very attractive for cancer targeted drug delivery where the use of antibodies is sometime compromised by the complicated processes adapted for antibody production and by the incapability to reliably produce antibodies under a strict quality control protocol required by the Food and Drug Administration (FDA).

Another advance that has been made in the past decades is the use of nanoparticles (NPs) as carriers for targeted drug delivery [16,17,18,19]. The high surface-area-to-volume ratio of NPs allows for delivering therapeutic agents to tumor sites by releasing them when- and where-ever needed. Thus, reduced dosage can be employed for treatment, helping to reduce the side effects. Among most NPs that have been tested so far, the super paramagnetic iron oxide NPs (SPIO-NPs) are very promising for targeted anti-cancer drug delivery. These NPs are biocompatible and are usually around 10–30 nm in diameter [20,21,22]. They have been widely used as FDA-approved contrast agents for magnetic resonance imaging (MRI) [23,24,25,26,27,28,29,30]. They have also been used for determining pharmacokinetics, which is the distribution and release of drugs after their administration. The drug loaded SPIO-NPs can be monitored through optical approaches for visualization and tracking of the drug delivery complexes *in vivo*, allowing physicians and researchers to further study and track the progress of the treatments.

The combination of RNA aptamer technique with NP-based drug delivery has shown remarkable success in reducing the side effects of chemotherapy in cancer treatment [12]. However, the immobilization of RNA aptamers on the surface of NPs is challenging due to the RNAs’ weak tolerance to chemical modifications. Unlike RNAs, DNAs are much more stable in most chemical reactions. Utilizing this advantage of DNAs, we designed a RNA-DNA hybrid aptamer in which a small fragment of single-stranded DNA with a sequence complementary to either the 3′ or 5′ end of an RNA aptamer is attached to the RNA aptamer without compromising its specificity for tumor cell surface antigens. The modification of these DNA linkers will allow the DNA-RNA hybrid aptamers to easily attach to NPs for targeted drug delivery. Since multiple DNA linkers can be added to one RNA aptamer, it is possible to endow RNA aptamers with multiple functions. For instance, the linkage of a fluorescent dye conjugated DNA linker and a binding group added DNA linker to an RNA aptamer will allow for the monitoring of drug targeting and releasing *in vivo*. In this study, we designed and tested a DNA-RNA hybrid aptamer that has a high affinity for the extracellular domain of the prostate-specific membrane antigen (PMSA) to improve the efficiency of the SPIO-NP-aptamer-based targeted drug delivery. The drug delivery platform consists of SPIO-NPs that are functionalized with carboxylic acid groups on their surface. These carboxylic acid groups provide anchor holds for streptavidin to be covalently bound to the SPIO-NPs through a peptide bond.

## 2. Results

### 2.1. The Formation of DNA-RNA Hybrid Aptamers for SPIO-NP-Based (Super Paramagnetic Iron Oxide NP-Based) Targeted Anticancer Drug Delivery

**Figure 1:**
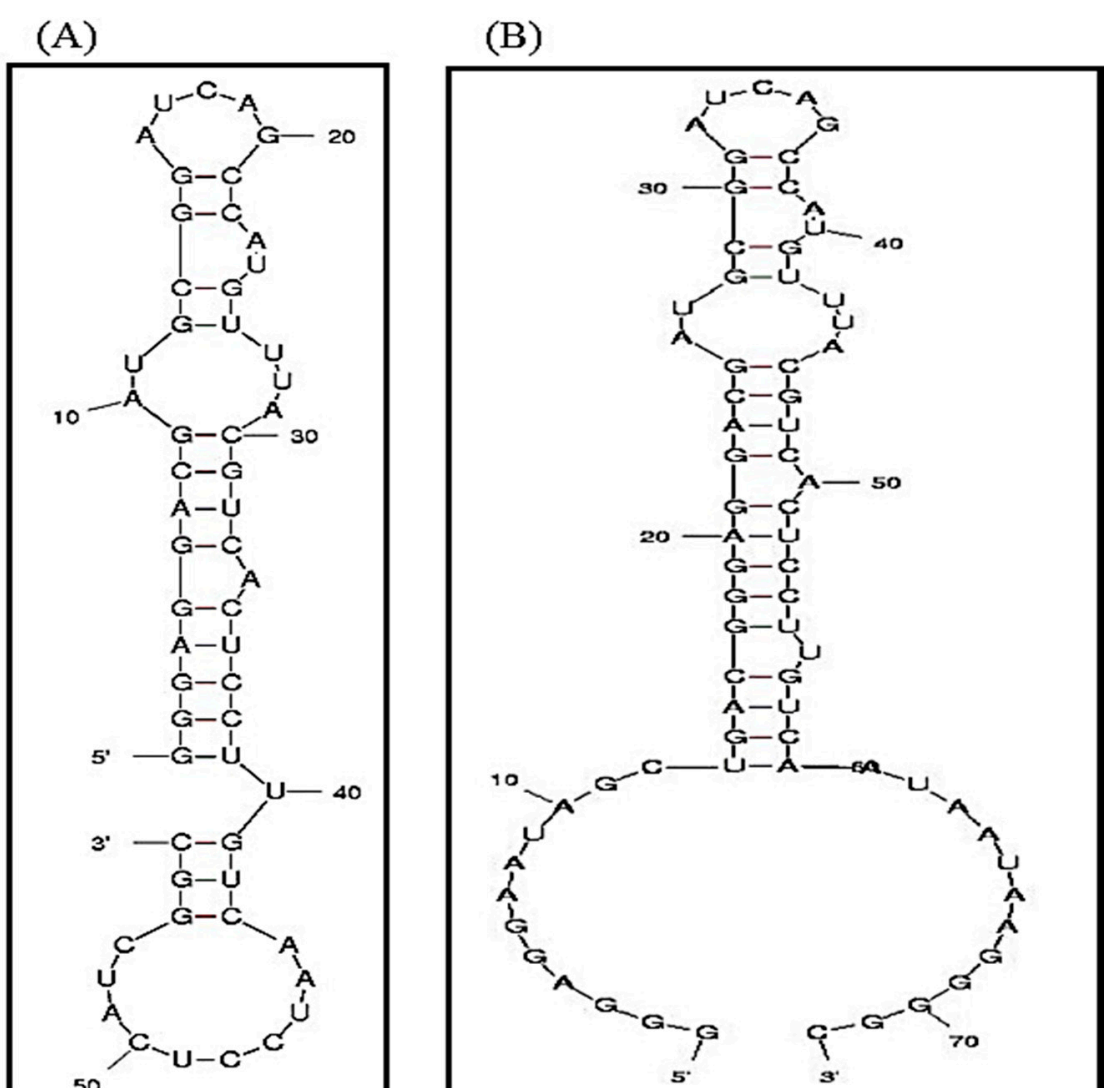
The secondary structures of (**A**) A10-3 and (**B**) A10-3-J1 aptamer, as predicted by the M-fold model. The highlighted region is the sites that are responsible for prostate-specific membrane antigen (PMSA) specificity. This structure is conserved in the A10-3-J1 aptamer to retain the functionality of the aptamer while providing the capability for DNA-RNA hybridization.

Biotin-streptavidin coupling is one of the most successful conjugation approaches for the surface functionalization of NPs. To utilize this mechanism for immobilizing RNA aptamers on NP surfaces, the biotinylation of RNA aptamers is required. This is, however, quite challenging due to the instability of RNA aptamers during biotinylation. Our study indicates that most RNA aptamers are degraded under the harsh biotinylation condition (data not shown). To overcome this difficulty, we redesigned the RNA aptamer by adding a DNA linker to the RNA aptamer to form a RNA-DNA hybrid aptamer. The advantage of this hybrid aptamer is that DNAs can be readily modified and functionalized, as compared to RNAs. This structure will endow additional functions to an RNA aptamer. For instance, the attachment of a DNA linker with a fluorescent dye will allow for fluorescence microscopy to monitor RNA aptamer entries into target cells. Figure 1B illustrates the design of our new aptamer, named A10-3-J1. This aptamer was designed based on A10-3, which is a widely used RNA aptamer for targeting PMSA^+^ prostate cancer cells [31,32]. The highlighted region of the aptamer shown in Figure 1A is responsible for PMSA specificity, as predicted by the M-fold model [33,34]. This region is conserved in the A10-3-J1 structure. The 3′ and 5′ of A10-3 are extended in such a way as to allow easy access for hybridization with a DNA linker, while conserving the specificity of the RNA aptamer for PMSA. Considering the drastic effects that a single base change in either of the aptamer ends could make on the secondary structure, this is a rather difficult and time-consuming process. To circumvent these difficulties, we used an iterative approach based on the M-fold model to conserve the folding of the specificity region. Modifications were continuously made until a single conformation thermodynamically stable was predicted for the given sequence, as shown in Figure 1B. This structure conserves the proper secondary structure for PMSA specificity and provides 12-base free RNA sequence on both the 3′ and 5′ ends of the aptamer for hybridization with complimentary DNA linkers. Thus, two DNA linkers can potentially be linked to one A10-3-J1 aptamer, making it possible for the aptamer to be functionalized in multiple ways.

**Figure 2:**
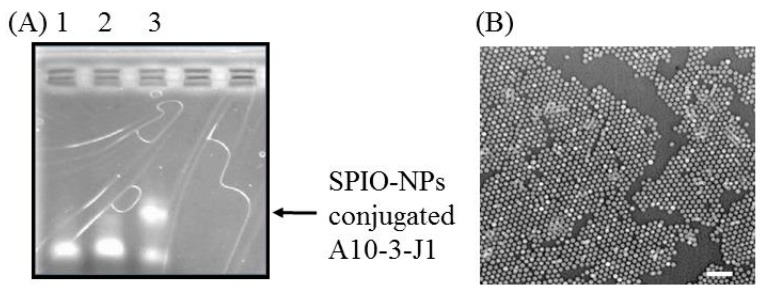
Determination of the conjugation of DNA-RNA hybrid aptamers to the super paramagnetic iron oxide NPs (SPIO-NPs) by electrophoresis and microscopic image of the nanoparticles. (**A**) Lane 1, A10-3-J1; Lane 2, DNA linker hybridized A10-3-J1 aptamer; and Lane 3, SPIO-NP conjugated A10-3-J1. The electrophoresis was run on a 2% agarose gel at 100 V for 30 min; (**B**) Microscopic image of the nanoparticles. Scale bar: 50 nm.

To prepare the DNA-RNA aptamer, we first synthesized an RNA aptamer from a double stranded DNA template using a Durascribe T7 RNA transcription kit with 2′ fluorine modified pyrimidines. The use of 2′-F modified pyrimidines for aptamer synthesis reduces chances of RNA degradation by RNase I [35]. The synthesized RNA aptamers were treated with DNase I to remove DNA templates. Before hybridization, the aptamers were folded into their proper secondary structures by being heated to 95 °C before they are placed on ice. After folding, biotinylated DNA linkers were mixed with the folded RNA aptamers to form DNA-RNA hybrid aptamers. The DNA-RNA hybrid aptamers were then conjugated to the streptavidin functionalized SPIO-NPs, as validated by electrophoresis (Figure 2A). The SPIO-NP was also imaged through transmission electron microscope analysis (Figure 2B). Finally, the doxorubicin was loaded to the aptamer-conjugated SPIO-NPs to form a drug delivery system.

### 2.2. Characterization of Specificity of the DNA-RNA Aptamers

**Figure 3:**
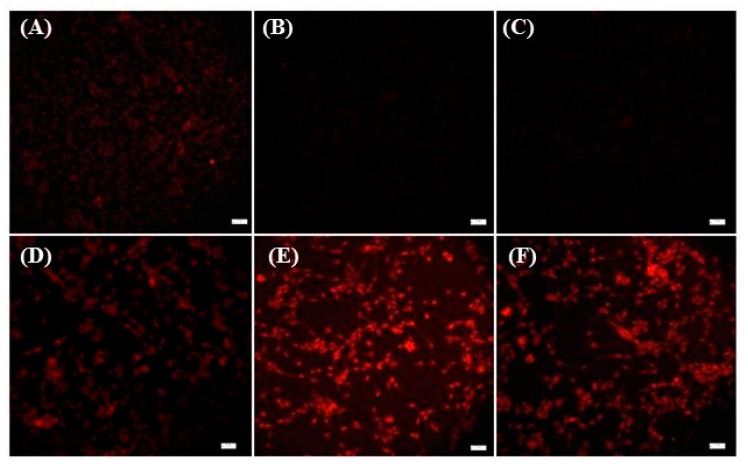
Determination of the specificity of DNA-RNA hybrid aptamers for PMSA. Free doxorubicin (1.5 µM) treated PC-3 (**A**); doxorubicin (1.5 µM) A10-3 conjugates treated PC-3 (**B**); doxorubicin (1.5 µM) A10-3-J1 conjugates treated PC-3 (**C**); free doxorubicin (1.5 µM) treated LNCaP (**D**); doxorubicin (1.5 µM) A10-3 conjugates treated LNCaP (**E**); and A10-3-J1 conjugates treated LNCaP (**F**). All images shown have the same cell seeding density. Fluorescence microscopy was performed at 2 h post treatment. Cells were washed with DPBS twice before performing the fluorescence microscopy. Scale bar: 20 µm.

To determine whether the DNA-RNA hybrid aptamer still retains its specificity for prostate specific membrane antigen (PSMA), we conjugated doxorubicin to the A10-3 and A10-3-J1 aptamers through the double helix of the DNA or RNA intercalation capability of the doxorubicin [36,37]. We chose doxorubicin because it is a very effective anticancer drug that has been widely used in cancer chemotherapy. However, its cytotoxicity leads to severe side effects during treatment. It is also a fluorescent anticancer drug, emitting red fluorescence at a wavelength of 590 nm when excited at 470 nm. Accordingly, the cellular delivery of doxorubicin can be detected through fluorescent microscopy. As shown in Figure 3A,D, the treatment of PC-3 and LNCaP cells with 1.5 µM of doxorubicin led to the entry of doxorubicin into the nuclei of both PMSA^−^ and PMSA^+^ prostate cancer cells. Nonetheless, the treatment of PMSA^−^ PC-3 cells with both doxorubicin-A10-3 and doxorubicin-A10-3-J1 conjugates showed significant blockage of the entry of doxorubicin into cell nucleus due to the absence of interaction between cellular antigen and A10-3-J1 to PC-3 cells (Figure 3B,F). The specific interaction between PMSA and the aptamers allows for efficient anticancer drug delivery to LNCap cells (Figure 3E,F). It seems that the doxorubicin-aptamer conjugates cannot penetrate cell membranes easily.

### 2.3. Targeted Delivery of Doxorubicin to PMSA^+^ (PMSA, Prostate Specific Membrane Antigen) Prostate Cancer Cells Using A10-3-J1 Conjugated SPIO-NPs

**Figure 4:**
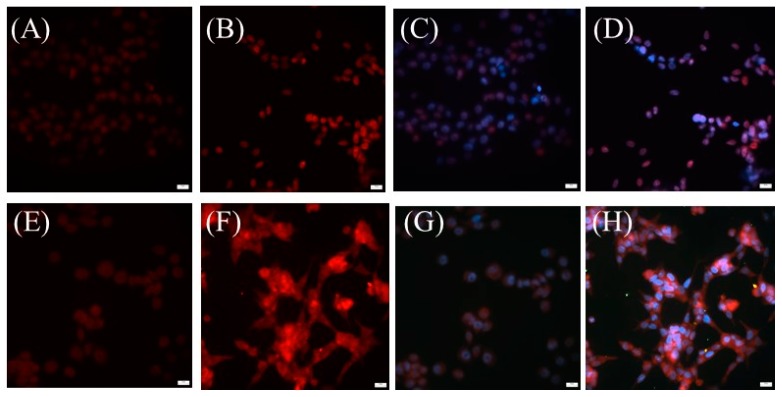
Cellular endocytosis of doxorubicin loaded SPIO-NPs. Both PC-3 and LNCaP cells were treated with either free doxorubicin (**A**)–(**D**) or doxorubicin loaded SPIO-NPs (**E**)–(**H**). (**A**), (**C**), (**E**), and (**G**): PC-3 cells; (**B**), (**D**), (**F**) and (**H**): LNCaP cells. (**C**), (**D**), (**G**), and (**H**): DAPI count-stained cells. Scale bar: 20 µm.

Next, we intended to determine whether the conjugated DNA-RNA hybrid aptamer to the SPIO-NPs will enhance the delivery of doxorubicin to the targeted prostate tumor cells and to avoid nonspecific drug delivery. To perform these tests, we first treated cells with free doxorubicin. As expected, doxorubicin diffused across the cell membrane and became concentrated in the cell nucleus. The overlapping of DAPI staining with the doxorubicin’s red fluorescence in the cell nucleus indicated the localization of doxorubicin in the cell nucleus (Figure 4C,D). However, this nuclear location of doxorubicin was not observed in doxorubicin loaded SPIO-NPs treated cells. As revealed by Figure 4G,H, doxorubicin appeared in both the cytoplasm and the nucleus compartment of the two distinct types of cells. The appearance of doxorubicin in the cytoplasm suggested that the SPIO-NP conjugated doxorubicin entered into the cells through endocytosis. Interestingly, doxorubicin loaded SPIO-NPs entered both PC-3 and LNCaP cells (Figure 4E,F). This unwanted nonspecific cellular entry could lead to severe side effects due to the cytotoxicity of doxorubicin.

**Figure 5:**
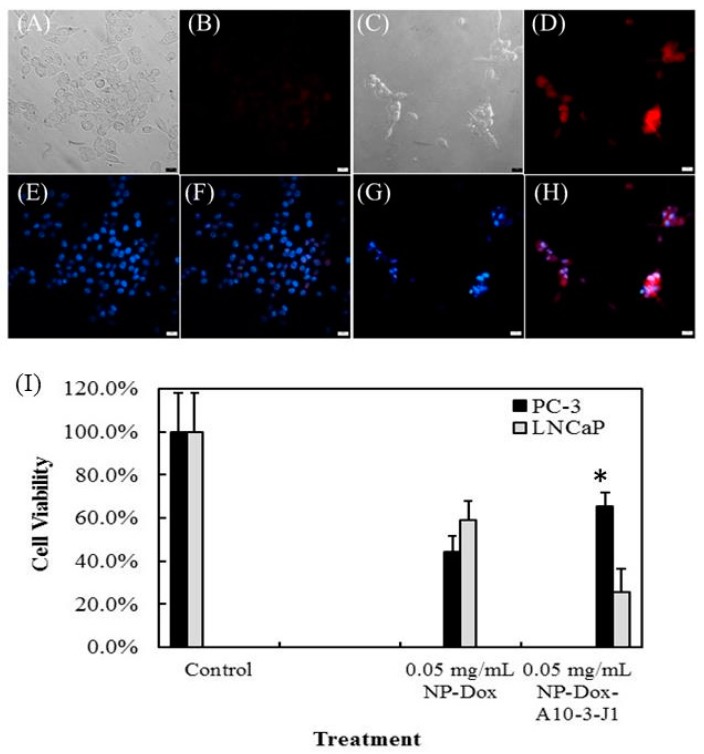
Fluorescent microscopy of targeted delivery of doxorubicin into PMSA^+^ prostate cancer cells using A10-3-J1 conjugated SPIO-NPs. All cells were treated for 2 h with 0.05 mg/mL A10-3-J1 conjugated SPIO-NPs loaded with doxorubicin, followed by wash in DPBS. Cells were fixed in 4% paraformaldehyde. The cells were count-stained with DAPI. Bright field micrograph images of PC-3 (**A**); doxorubicin loaded A10-3-J1-SPIO-NPs treated PC-3 (**B**); bright field micrograph images of LNCaP (**C**); doxorubicin loaded A10-3-J1-SPIO-NPs treated LNCaP (**D**); DAPI counted-stained PC-3 (**E**); DAPI and doxorubicin composite images PC-3 (**F**); DAPI counted-stained LNCaP (**G**); and DAPI and doxorubicin composite images LNCaP (**H**). Scale bars: 20 µm. (**I**): cell viability after treatment with doxorubicin loaded A10-3-J1 conjugated SPIO-NPs. Error bars are representative of the standard deviation for each individual treatment. * *p*-value < 0.05.

As shown in Figure 3, the intercalation of doxorubicin into A10-3-J1 aptamer prevents the entry of doxorubicin into PMSA^−^, *i.e.*, nontargeted cells. Nevertheless, doxorubicin delivered using the aptamer-doxorubicin approach will be very limited, as only one molecule of doxorubicin can be intercalated into one molecule of aptamer. This limitation, however, can be overcome using nanoparticles, as more doxorubicin can be loaded to one nanoparticle due to the nanoparticle’s superior surface-area-to-volume ratio. To test this feasibility, we loaded doxorubicin to A10-3-J1 conjugated SPIO-NPs through its hydrophobic interaction with the remaining free carboxyl groups on the NPs. Thus, the doxorubicin can be released from NPs inside the endosomes due to their acidic environment. Furthermore, the treatment of PMSA^+^ LNCaP cells with doxorubicin loaded A10-3-J1-SPIO-NPs led to the deaths of most of the cells (Figure 5C,D,G), while most of the PMSA^−^ PC-3 cells survived after treatment (Figure 5A,B,F). The dual color fluorescence microscopy indicated that only very few doxorubicin loaded A10-3-J1-SPIO-NPs entered into PMSA^−^ PC-3 cells (Figure 5F), whereas most doxorubicin loaded A10-3-J1-SPIO-NPs entered into PMSA^+^ LNCaP cells (Figure 5H). Clearly, the conjugation of A10-3-J1 aptamers to the NPs prevents the entry of NPs into PMSA^−^ cells. This is consistent with experimental results shown in Figure 3 where the doxorubicin is intercalated into an A10-3-J1 aptamer. Taken together, we stipulated that the functionalization of NPs with aptamers prevented the entry of doxorubicin-SPIO-NPs conjugates into non-targeted cells. It is clear that doxorubicin-A103-J1 conjugates can only be delivered into targeted cells.

The MTT assay further confirmed this observation. As shown in Figure 5I, the survival rate of both PC-3 and LNCaP were only marginally different after treatment with doxorubicin loaded NPs. The cell survival rate of PC-3 cells was about 44.2%, whereas it was 58.8% for LNCaP cells after treating with doxorubicin loaded NPs. However, the survival rate of PC-3 increased to 65.3% after being treated with doxorubicin loaded A10-3-J1-SPIO-NPs, suggesting that cells are protected from cytotoxic doxorubicin when aptamers are used. In contrast, the cell survival rate of LNCaP was only about 25.7% after being treated with doxorubicin loaded A10-3-J1-SPIO-NPs.

## 3. Discussion

We have demonstrated that the adverse effects of doxorubicin can be significantly reduced, or even eliminated, by means of a drug delivery system developed in this work. Our experimental results indicate the remarkable death of PMSA^+^ prostate cancer cells, suggesting targeted doxorubicin delivery into prostate cancer cells when aptamers and NPs are used for delivery. In our test, the fluorescence intensity of NP-loaded doxorubicin was measured to ensure that the same amount of doxorubicin is used in all experiments. The experimental results demonstrated that the drug delivery platform design enhanced specificity and cytotoxicity, leading to a two-fold PSMA^+^ cell death, compared to previously reported results where nanoparticle-mediated drug delivery was not used [36]. SPIO-NP has been employed to conjugate with DNA oligos for targeted delivery to human colon carcinoma cells. It has been found that the internalization and biological activity of the NPs depend on the oligonucleotide’s density [38]. *In vivo* imaging using aptamer-SPION and Epirubicin-5TR1, has been reported for treating colon cancer cells. A high level of NP accumulation was found in the tumor site, confirming targeted delivery using aptamer conjugated SPIONs [39]. A thermally cross-linked SPION containing PSMA aptamers has been also examined in a mouse tumor model, suggesting that this type of NP has potential in *in vivo* imaging [40]. Furthermore, an A10 RNA aptamer targeting PSMA^+^ cells and a peptide aptamer targeting PSMA^−^ cells was conjugated to SPION NPs. Their selective uptakes and effective drug delivery were verified by Prussian blue staining and trypan blue staining [41]. Our study further confirmed the effectiveness of the aptamer for selective drug delivery. In addition, the *in vivo* stability of a biotin-DNA linker is critical to its clinical application. While we did not characterize the stability of biotin-DNA linkers *in vivo*, experimental results acquired by other groups suggested the *in vivo* stability of a biotin-DNA linker [42]. On the other hand, it might be critical to determine the dissociation constant of aptamer against PSMA. Taken together, we developed an A10-3-J1 PSMA specific aptamer and hybridized DNA-RNA to strongly conjugate nanoparticle and anticancer agent for functionalization of targeted drug delivery platform.

## 4. Experimental Section

### 4.1. Nanoparticles

The SHS-010 SPIO-NPs were prepared by Ocean Nanotech (Fayetteville, AR, USA). These unique iron oxide (Fe_3_O_4_) NPs, about 10 nm in diameter, are functionalized by coating with a polymer that adds carboxyl groups to the surface of NPs. These surface carboxyl groups are then used to form a peptide bond with streptavidin for binding with biotinylated DNA-RNA hybrid aptamers. Once the streptavidin is added to the NPs, the suspension is mixed with doxorubicin (Sigma, St. Louis, MO, USA) to load the drugs to the NPs. Doxorubicin binds to the NPs through hydrophobic interactions with the remaining free carboxyl groups on the NPs, creating a pH-sensitive doxorubicin delivery platform.

### 4.2. Aptamer Synthesis

The A10-3-J1 dsDNA was PCR amplified using a pair of primes 5′-TAATACGACTCACTATAGGGAGGAATAGCT-3′, and 5′-GCCCCTTATTATTGACAAGGAGTGACGTAA-3′. The thermal cycler was set to run 25 cycles with the following conditions: denaturation—30 s at 94 °C, annealing—30 s at 60 °C, polymerization—30 s at 72 °C; last cycle of denaturation—1 min at 94 °C, last cycle of polymerization—4 min at 72 °C. The fragments were gel-purified after PCR. The purified dsDNA was then transcribed to RNA using a Durascribe T7 Transcription kit (Epicentre Biotechnologies, Madison, WI, USA) with 2′ fluorine modified pyrimidines. After transcription, the solution was treated with DNase I, and the RNA was collected using a nucleotide removal kit (Qiagen, Redwood, CA, USA). The sequence of transcribed A10-3-J1 ssRNA is as follows: 5′-GGGAGGAAUAGCUGACGGGAGGACGAUGCGGAUCAGCCAUGUUUACGUCACUCCUUGUCAAUAAUAAGGGGC-3′.

### 4.3. Conjugation of DNA-RNA Aptamers to the SPIO-NPs and Loading the Doxorubicin to the Aptamer-Conjugated SPIO-NPs

To attach the RNA aptamers to the streptavidin functionalized SPIO-NPs, a DNA linker was designed as follows: A10-3-J1 5′ DNA Linker: 5′-GCTATTCCTCCCTATATG-Biotin-3′. After hybridization, which details are described in the Results section, the RNA-DNA aptamers were linked to the SPIO-NPs through binding between streptavidin and biotin. Hybridization and attachment of DNA-RNA aptamers to the SPIO-NPs was verified by agarose gel electrophoresis.

### 4.4. Cell Culture

PSMA^−^ PC-3 (CRL-1435, American type culture collection, Manassas, VA, USA) and PSMA^+^ LNCaP (CRL-1740, American type culture collection, Manassas, VA, USA) prostate cancer cell lines were used to demonstrate the targeted drug delivery system developed in this work. The complete growth medium for PC-3 cells included F12-K (American type culture collection, Manassas, VA, USA) supplemented with 10% fetal bovine serum (FBS) (American type culture collection, Manassas, VA, USA) and penicillin/streptomycin (Mediatech, Manassas, VA, USA), whereas the complete growth medium for LNCaP cells was RPMI 1640 (American type culture collection, Manassas, VA, USA) supplemented with 10% FBS and penicillin/streptomycin.

### 4.5. Fluorescence Microscopy

To determine the delivery of doxorubicin into prostate tumor cells through fluorescent microscopy, cells were seeded in 1.6 × 10^4^ cells per well (70% confluence) in a cell culture chamber slide (Lab-Tek, Hatfield, PA, USA) supplied with 300 µL of the complete growth medium for the respective cell line. The slide was incubated at 37 °C for 24 h in a humidified 5% CO_2_ atmosphere prior to experiments. To test the SPIO-NP-Aptamer targeted drug delivery system, the medium was aspirated out of the chamber, followed by adding with 300 µL of complete growth media containing free doxorubicin, NP-doxorubicin conjugates, or SPIO-NP-Aptamer-doxorubicin constructs to the chamber. The slide was then placed back into the incubator and cultured for 2 h before fixation. To fix the cells, the medium was removed from the chamber, followed by washing once with 300 µL of room temperature DPBS (Mediatech, Manassas, VA, USA) and the addition of 150 µL of room temperature 4% freshly-made paraformaldehyde (Sigma, St. Louis, MO, USA). The slide was allowed to incubate at room temperature for 10–20 min, followed by a brief rinse with 100 µL of DPBS. The DAPI (Research Organics, Cleveland, OH, USA) was applied to stain the cell nucleus by replacing the DPBS with 300 µL of 300 nM DAPI dye solution in DPBS. Afterwards, the slide was incubated at 37 °C for 1–5 min before being washed twice with 150 µL of room temperature DPBS. A portion of 100 µL of DPBS was added to the chamber after washing to prevent the slide from drying out during fluorescence microscopy. An inverted fluorescence microscope (IX71, Olympus, Center Valley, PA, USA) equipped with a CCD camera (Retiga 4000R, Q-Imaging, Bullhead, AZ, USA) was used to examine cells inside the chamber slide. Rhodamine filter (Chroma Technology Corp., Bellows Falls, VT, USA) was used for detecting intracellularly delivered doxorubicin.

### 4.6. Cell Viability and Cytotoxicity Evaluation

To interrogate the potential apoptotic effects of delivered drugs, MTT assays were performed using a cell growth viability kit (Biotium, Hayward, CA, USA). Cells were seeded at 1.3 × 10^4^ cells per well (70% confluence) in a 96-well plate (Falcon, San Jose, CA, USA). Each well was filled with 100 µL of the complete growth media for the respective cell lines. The 96-well plate was incubated under a 5% CO_2_ atmosphere at 37 °C for 24 h. The medium was then removed from the wells, and a portion of 100 µL of the complete medium containing free doxorubicin, NP-doxorubicin conjugates, or SPIO-NP-Aptamer-doxorubicin constructs was added to each well. Cell viability was determined using the MTT kit by following an instruction given by the manufacturer. The MTT assay was read using a microplate reader (Power Wave XS, Bio-Tek, Winooski, VT, USA) at a test wavelength of 570 nm and a reference wavelength of 630 nm.

### 4.7. Statistical Analysis

All experiments were carried out in at least triplicate. The means and standard deviations of experimental results were computed and a *p-*value of 0.05 or less was considered to be a statistically significant difference between two groups of experiments.

## 5. Conclusions

We developed an A10-3-J1 PSMA specific aptamer and hybridized DNA-RNA to strongly conjugate SPIO nanoparticle and doxorubicin anticancer agent for targeted drug delivery. The DNA RNA hybridized aptamer antitumor agent-conjugated nanoparticle was able to enhance the cytotoxicity of targeted cells while minimizing collateral damage to non-targeted cells. It has specificity to PSMA^+^ prostate cancer cells. Aptamer inhibited nonspecific uptake of membrane-permeable doxorubic to the non-target cells, leading to reduced untargeted cytotoxicity and endocytic uptake while enhancing targeted cytotoxicity and endocytic uptake. The experimental results indicate that the drug delivery platform can yield statistically significant effectiveness being more cytotoxic to the targeted cells as opposed to the non-targeted cells.
